# Exploring Transformer and Graph Convolutional Networks for Human Mobility Modeling

**DOI:** 10.3390/s23104803

**Published:** 2023-05-16

**Authors:** Riccardo Corrias, Martin Gjoreski, Marc Langheinrich

**Affiliations:** Computer Systems Institute, Faculty of Informatics, Università della Svizzera italiana (USI), 6900 Lugano, Switzerland; riccardo.corrias@usi.ch (R.C.); marc.langheinrich@usi.ch (M.L.)

**Keywords:** machine learning, deep learning, graph convolutional networks, transformers, mobility modeling

## Abstract

The estimation of human mobility patterns is essential for many components of developed societies, including the planning and management of urbanization, pollution, and disease spread. One important type of mobility estimator is the next-place predictors, which use previous mobility observations to anticipate an individual’s subsequent location. So far, such predictors have not yet made use of the latest advancements in artificial intelligence methods, such as General Purpose Transformers (GPT) and Graph Convolutional Networks (GCNs), which have already achieved outstanding results in image analysis and natural language processing. This study explores the use of GPT- and GCN-based models for next-place prediction. We developed the models based on more general time series forecasting architectures and evaluated them using two sparse datasets (based on check-ins) and one dense dataset (based on continuous GPS data). The experiments showed that GPT-based models slightly outperformed the GCN-based models with a difference in accuracy of 1.0 to 3.2 percentage points (p.p.). Furthermore, Flashback-LSTM—a state-of-the-art model specifically designed for next-place prediction on sparse datasets—slightly outperformed the GPT-based and GCN-based models on the sparse datasets (1.0 to 3.5 p.p. difference in accuracy). However, all three approaches performed similarly on the dense dataset. Given that future use cases will likely involve dense datasets provided by GPS-enabled, always-connected devices (e.g., smartphones), the slight advantage of Flashback on the sparse datasets may become increasingly irrelevant. Given that the performance of the relatively unexplored GPT- and GCN-based solutions was on par with state-of-the-art mobility prediction models, we see a significant potential for them to soon surpass today’s state-of-the-art approaches.

## 1. Introduction

The estimation of human mobility patterns is widely recognized as being vital for the provision of future services and solutions [1]. Example application domains include digital solutions for more effective advertising [2], enhanced security [3], improved urban services [4], and tracking the spread of a contagious disease (e.g., COVID-19) [5]. Mobility modeling is also essential for behavioral tracking and interventions related to mental health and well-being [6,7], given that different symptoms (e.g., increased vs. decreased mobility and social interactions) can be connected to mental health problems, such as depression [8].

Mobility modeling is a well-established research domain. In the past, the focus has been on utilizing Markov models to analyze frequent mobility patterns [9,10,11]. Machine learning (ML) techniques—including Decision Trees [12], Support Vector Machines [13], and Random Forest [14]—are some of the ML algorithms that have been used in addition to Markov-based approaches to predict future mobility patterns. The first ML methods relied on features (e.g., time- and frequency-based descriptors) that were taken from the mobility trajectories [15]. The more recent ML methods replaced the more traditional feature- and Markov-based approaches by utilizing raw trajectory data in combination with end-to-end learning (e.g., Recurrent Neural Networks—RNNs [16,17], and Long Short-term Memory networks—LSTMs [18]).

Unlike the existing mobility modeling methods based on RNNs and LSTMs, this study explores how two recent Deep Learning methods—General Purpose Transformer (GPT) and Graph Convolutional Networks (GCNs)—perform on next-place prediction tasks. GPT-based and GCN-based models have recently achieved outstanding results in image processing (e.g., diffusion-based models [19]) and natural language processing (e.g., ChatGPT [20]). Our reason for applying GPT-based models to mobility data is the similarity between sentences and movement trajectories. GPTs were initially applied in the NLP domain, where the words in each sentence are processed as input sequences. Similarly, locations that constitute the movement trajectories can be processed as “input words” to a GPT model. Additionally, the task of next-place prediction is quite similar to the task of next-word prediction, which is often used for the pre-training of Large Language Models (LLMs) [20]. On the other hand, the task of next-place prediction can be viewed as a graph problem where each node represents the places visited at a specific moment. By using a GCN-based approach to model this data, we try to model hidden interdependencies between the nodes. However, it is unclear how GPT- and GCN-based models will perform in the context of next-place prediction. Some specific challenges explored in this study are the model behavior on a small vs. a big dataset and the behavior on a sparse vs. a dense dataset. Our study contributions thus include the following:

- Developing GPT- and GCN-based architectures for next-place prediction.
- Adapting the popular Flashback-LSTM model for next-place prediction to our experimental setup to serve as a state-of-the-art baseline.
- Pre-processing three real-life datasets for mobility modeling (both sparse and dense) and performing a comparative analysis between GPT, GCN, and Flashback-LSTM by taking into account the behavior of the models with respect to the size of the datasets (e.g., small vs. big datasets) and the behavior of the models on a sparse vs. a dense dataset. The code for our experiments is publicly available (https://github.com/corrir/Transformers-and-Graph-Convolutional-Networks-for-Human-Mobility-Modeling (accessed on 16 March 2023)).

The rest of this paper is structured as follows. Section 2 summarizes the relevant related work on human mobility models. Section 3 describes the three datasets used in the study, including the pre-processing steps for each dataset. Section 4 details the three methods used in our experiment (a GPT-based model, a GCN-based model, and Flashback-LSTM). The experimental setup and results are presented in Section 5. We discuss our findings and conclude the study in Section 6.

## 2. Related Work

**Figure 1:**
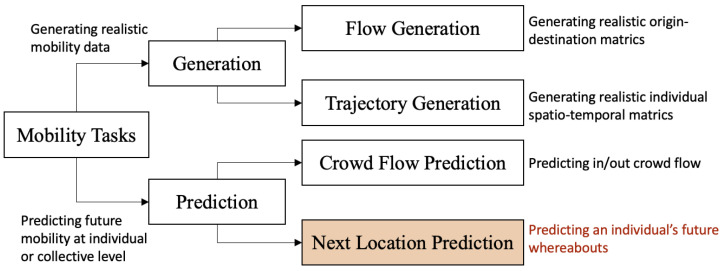
Mobility-task taxonomy. Based on the work by Luca et al. [21].

When reviewing the state-of-the-art deep learning models for mobility tasks, Luca et al. [21] proposed a two-level taxonomy (see Figure 1). At the first level, mobility tasks are divided into *generative tasks* and *predictive tasks*. At the second level, the *generative* tasks are split into *flow generation* and *trajectory generation*, while the *predictive* tasks are split into *crowd-flow prediction* and *next-location prediction*. Next-location prediction focuses on estimating an individual’s future movements given the historical movement of that individual. We present a more detailed analysis of this task in the following subsection. On the other hand, crowd flow prediction estimates aggregated traffic flows [22] and the demand for shared transportation tools (e.g., city parking [23] and bike-sharing [24]).

### Next-Place Prediction

Human mobility models aim to predict future places visited by individuals or groups. Next-place predictors focus on individual predictions and use previous observations to anticipate an individual’s future location. Next-place prediction can be helpful for various purposes, such as monitoring public health [25], well-being [26], as well as improving travel recommendations, geomarketing, and social network link predictions [27]. These predictions can also be used by authorities to enhance public transportation, urban planners to plan for the future growth of a city, and transportation companies to provide better traffic management services.

Next-place prediction is a complex task, because it requires the understanding of an individual’s unique routine and the consideration of various factors influencing human mobility. Luca et al. [21] defined it as follows:

“Next-location predictors forecast an individual’s future whereabouts, based on their historical observations... Formally, let *u* be a user, $T_{u}$ their trajectory, and $p_{t}\in T_{u}$*u*’s current location, next-location prediction aims at predicting *u*’s next destination $p_{t+1}$. This problem is treated in two ways: (i) as a multiclass classification task, in which we have as many classes as locations and we aim at predicting the next visited location $p_{t+1}$; or (ii) as a regression task, predicting $p_{t+1}=\left(x_{t+1},y_{t+1}\right)$, where $x_{t+1}$ and $y_{t+1}$ are the next location’s geographic coordinates. A variant of next-location prediction aims at forecasting the next Point Of Interest (POI) $p_{t+1}$ an individual *u* will visit given their trajectory $T_{u}$. Regardless of the specific definition, next-location predictors output a ranking of the probability of each location to be *u*’s next destination.”

The initial methods for next-place prediction were based on probability or patterns. These methods could handle datasets of moderate size [28] but required significant effort in feature engineering and could not capture long-term temporal and geographical correlations. One example of a probability-based method combined multiple features of trajectories, such as the spatiotemporal, semantic characteristics of the modeled places and the distance traveled [29]. Other methods use stochastic models to cluster geographical coordinates into relevant areas [9], or create a Mobility Markov Chain in which each Markov state represents a modeled place, and the transitions between the Markov states correspond to movements between POIs [30]. Another example, the *Wherenext* system [12], uses patterns to characterize mobility as a sequence of frequently visited places, considering the duration of the journey.

More recent approaches utilize deep learning techniques to overcome traditional methods’ limitations by capturing temporal, geographical, and social–geographic patterns in large datasets. These approaches use mechanisms such as RNNs, LSTM, gated recurrent units (GRUs), fully connected layers (FCs), attention mechanisms, and Convolutional Neural Networks (CNNs). For example, DeepMove [16], RNN+SAtl [17], and Flashback [18], are deep learning architectures that learn time and location embeddings and apply RNNs, including LSTMs, to predict the future places that will be visited by a user, i.e., next-place prediction.

Unlike the existing mobility modeling methods based on RNNs and LSTMs, this study explores GPTs and GCNs for next-place prediction. A recent review study by Luca et al. [21] analyzed the existing deep learning approaches for human mobility modeling. Their analysis showed that, out of the 240 studies, only three were related to GPT- and GCN- based modeling. From those three studies, the MVGCN (Multiview Graph Convolutional Network) [31] and SI-GCN (Spatial Interaction GCN) [32] are two GCN-based networks used for flow generation. Given that flow generation is a task for estimating the flows between a group of geographic places based on those locations’ data (such as population, POIs, land use, and distance to other sites), it is a separate task from next-place prediction. Finally, only one study presented a transformer-like architecture, the DWSTTN (Deep Wide Spatio Temporal Transformer Network) [33]. The DWSTTN uses historical pick-up and drop-off data from taxi companies in Porto and Manhattan to predict a taxi’s next destination. The scarcity of GPT- and GCN-based modeling in the related work is not surprising, given that these approaches are relatively new. Thus, to the best of our knowledge, we present the first study to compare GPT-based and GCN-based models for next-place prediction.

## 3. Datasets

Mobility datasets can be collected using various methods, including GPS tracking, mobile phone data, or surveys. The data can be collected at different levels of granularity, ranging from the detailed tracking of individual movements to more aggregated data on the movements of groups or populations. In this study, we worked with three datasets: *Foursquare*, *Gowalla*, and *Breadcrumbs*. All three datasets provide the following information:

- User ID: This feature is a unique identifier for each individual or entity represented in the dataset.
- POI ID: This feature is a unique identifier for each Point of Interest (POI) represented in the dataset.
- Latitude: This feature represents a value to identify the position of the POI. More particularly, it represents the North–South position (vertical).
- Longitude: This feature represents a value to identify the position of the POI. More particularly, it represents the East–West position (horizontal).
- Timestamp: This feature answers the question of *when* a specific user is in a certain POI.

### 3.1. Foursquare Dataset

**Figure 2:**
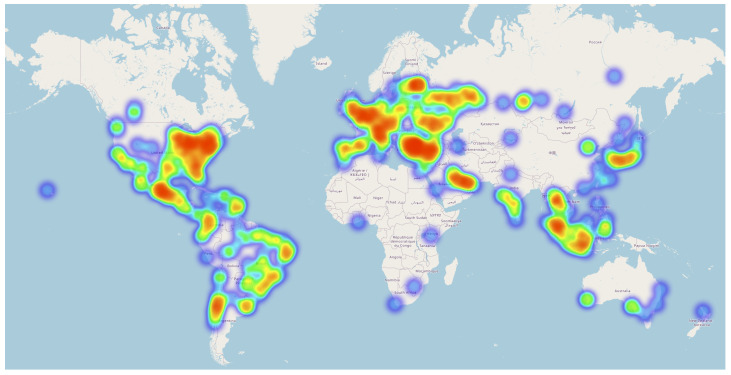
Distribution of user locations available in the Foursquare dataset. Red represents higher density and blue represents lower density.

Foursquare was a company that provided location-based services and data through its mobile and web applications. The *Foursquare* dataset [34,35] includes data on the locations of millions of businesses, venues, and points of interest around the world, as well as data on the movements and activities of users who have checked in at these locations using the Foursquare services. This dataset contains Foursquare check-in information gathered over an extended period (about 18 months, from April 2012 to September 2013). It includes check-ins totaling 33,278,683 from 266,909 people in 3,680,126 locations in 415 cities across 77 countries. These 415 cities have at least 10,000 check-ins apiece, making them the 415 most frequented towns across the globe. Figure 2 depicts the distribution of user locations available in the dataset.

### 3.2. Gowalla Dataset

Similar to the Foursquare dataset, the *Gowalla* dataset was extracted from another location-based service (Gowalla). From February 2009 to October 2010, 6,442,890 check-ins from Gowalla users were gathered using the service’s API [36]. Compared to the *Foursquare* dataset, *Gowalla* has a smaller number of check-ins.

### 3.3. Breadcrumbs Dataset

The *Breadcrumbs* dataset was collected in a 12-week study by Moro et al. [37] from 81 users in Lausanne, Switzerland using several mobile phone sensors (GPS, WiFi, Bluetooth). Compared with the other two datasets (Foursquare and Gowalla), Breadcrumbs is based on continuous mobile sensing (e.g., 1 Hz GPS data), which is different than the sparse, check-in-based data available in Foursquare and Gowalla.

In our earlier study on mobility modeling, we used the hierarchical spatiotemporal DBSCAN, a clustering algorithm, to determine POIs in the Breadcrumbs dataset, i.e., the shared locations where users spend the majority of their time [38]. The algorithm employed the following parameters: a maximum cluster radius of 250 m and a waiting time of 5 min (stop-points with a shorter waiting time were ignored). After POI extraction, we subsampled the dataset at a $15-min rate (i.e., we would get check-in information for each user every 15 min) to make it more comparable to the check-in-based datasets. This led to 15,898 check-ins from 81 users in 40 different places.

### 3.4. Pre-Processing

We performed several pre-processing steps for each of the three datasets, *Foursquare*, *Gowalla*, and *Breadcrumb*, in order to bring them to a similar data format.

- **Cleaning and standardization**:
  We noticed that not all timestamps followed the same format. Since there can be a variety of Timestamp (Date), we decided to transform all Timestamp fields into the following format:
  yyyy−MM−ddTHH:mm:ssZ
  2013−03−12T20:34:32Z
  Another step done for data cleaning was the elimination of lower-quality data. Following the related work [18], we decided to remove all the users with less than 100 check-ins and POIs that were visited less than 50 times from *Foursquare* and *Gowalla*. The last step regarding data transformation was standardizing all the *userIDs*, i.e., we mapped all string-based *userIDs* into unique integers.
- **Additional information**: To give the models more information for each check-in and improve the predictions’ accuracy, we added a feature to identify the day of the week on which a specific check-in was registered.

## 4. Machine Learning Methods

### 4.1. Flashback-LSTM

Flashback-LSTM was introduced by Yang et al. [18] to model sparse user mobility data by exploiting flashbacks on hidden states in RNNs. Sparse user mobility is an important challenge for check-in-based datasets, given that vanilla RNNs have been created to learn from continuous and equidistant sequences (e.g., sentences), and hence cannot handle sparse data easily. To address the challenge, Flashback uses the spatiotemporal context by searching for previous hidden states with strong predictive potential similar to the current environment, i.e., the instance to be predicted, given a user’s mobility trace represented as a sequence of POIs:

$$\left\{\cdots ,P_{i-3},P_{i-2},P_{i-1},P_{i},P_{i+1},\cdots \right\}$$

The temporal and spatial difference between two check-ins, $P_{i}$ and $P_{j}$, is denoted as $\Delta T_{i,j}$ and $\Delta D_{i,j}$. To predict the subsequent position $p_{i+1}$ Flashback LSTM leverages the spatiotemporal context by computing the weighted average of the historical hidden states with the weight $W(\Delta T_{i,j},\Delta D_{i,j})$ as an aggregated hidden state to seek prior hidden states rather than relying on the present hidden state $h_{i}$ to predict the subsequent position $p_{i+1}$. Flashback-LSTM also learns user-specific embedding vectors to represent their preferences. This vector is then concatenated with the combined hidden state and put into a fully connected layer for location prediction.

In our experiments, Flashback-LSTM served as a state-of-the-art baseline model. For the training procedure, we used the Cross-Entropy Loss Function in order to adjust the model weights. As an optimization algorithm, we used Adam, and the learning rate used throughout the experiments was dynamically adjusted, starting from a value of 0.01. Every 20 epochs, a gamma value of 0.2 was applied to achieve better convergence to the prediction. The whole training phase was done for 100 epochs.

### 4.2. Multivariate Time Series Graph Neural Network (MTGCN)

Multivariate time series forecasting can be viewed as a graph problem, where in our specific task of next-place prediction, the variables in the time series are represented as nodes. In other words, each node represented the POIs visited at a specific moment. The assumption is that the nodes are connected through hidden interdependencies. By using graph neural networks to model this data, it is possible to preserve the temporal trajectory of the time series while also leveraging the interdependencies between the variables.

Our GCN model was based on the Multivariate Time Series Graph Neural Network (MTGNN) proposed by Wu et al. [39]. GNNs are particularly adept at handling dependencies between entities. However, GNNs need a clear graph structure for information propagation to perform effectively. This means they cannot be used directly on multivariate time series data, where the dependencies between variables are not known in advance. The suggested method automatically extracts the unidirected relationships between variables through a graph learning module. External knowledge, such as variable qualities, may be included in the graph learning module. A mix-hop propagation layer and a dilated inception layer are suggested as two new layers to capture spatial and temporal relationships in the time series data. These layers are collectively taught inside an end-to-end architecture, together with the graph learning, graph convolution, and temporal convolution modules.

**Figure 3:**
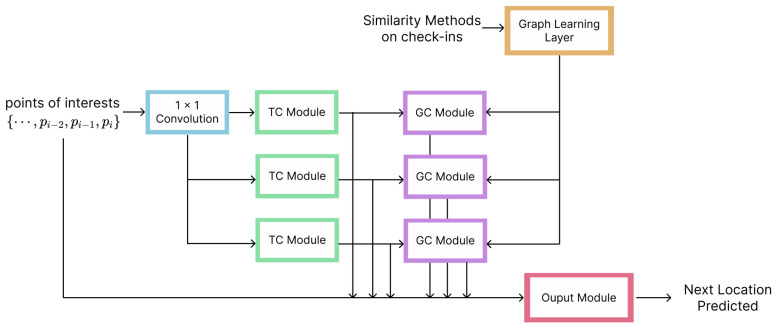
Architecture overview. Multivariate Time Series Graph Neural Network for next-POI prediction.

When using the MTGNN (Figure 3) for next-location prediction, the sequence of visited POIs is initially projected onto a latent space using a 1×1 standard convolution. To capture geographical and temporal relationships, graph convolution modules and temporal convolution modules are interleaved with one another. The hidden graph adjacency matrix, which is used by the graph convolution modules, is learned by the graph learning layer through the use of similarity methods between a portion of the different check-ins for each user in each minibatch. Finally, to obtain the predicted next location, the output module projects hidden characteristics to the specified dimension.

More precisely, the problem can be formulated as follows:

Let $z_{t}\in R^{n}$ denote the value of a multivariate variable of dimension *N* at time step *t*, where $z_{t}\left[i\right]\in R$ denotes the value of the *i*th variable at time step *t*. Given a sequence of historical *P* time steps of observations on a multivariate variable, we have

$$X=\{z_{t_{1}},z_{t_{2}},\cdots ,z_{t_{P}}\}$$

The goal is to predict the value:

$$Y=z_{t_{P+1}}$$

The input signals (e.g., historical check-ins) can be combined with other information, such as the day of the week and the time of day. We view variables in multivariate time series as nodes in graphs from a graph-based approach. The graph adjacency matrix, which is generated by the Graph Learning Layer in MTGNN, defines the connections between the nodes. The adjacency matrix may be defined as follows:

$$A\in R^{N\times N}$$

with:

$$\begin{array}{cc} A_{ij} & =c>0\;\mathrm{if}\;(v_{i},v_{j})\;\mathrm{are}\;\mathrm{connected}\;\mathrm{by}\;\mathrm{an}\;\mathrm{edge}\\ A_{ij} & =0\;\mathrm{if}\;(v_{i},v_{j})\;\mathrm{are}\;\mathrm{not}\;\mathrm{connected}\;\mathrm{by}\;\mathrm{an}\;\mathrm{edge} \end{array}$$

A Graph Learning Layer, a Graph Convolution Module, Temporal Convolution Module, and an Output Module are the particular parts that comprise the MTGNN. The Graph Learning Layer computes a graph adjacency matrix, which is subsequently utilized as input for the Graph Convolution Module to find hidden relationships between the nodes. These modules are interleaved with the Temporal Convolution Module, which captures temporal relationships. To have a better and clear understanding of how the MTGNN works, we can go through the different layers and modules:

- **Graph Learning Layer**: Different from many Graph Neural Networks that can only operate with a known graph structure (which is not the case with time series forecasting because the structure is unknown before the training procedure), the MTGNN uses this layer to extract a sparse graph adjacency matrix depending on the data. Previous research has measured the similarity between pairs of nodes using distance metrics, such as the dot product and Euclidean distance, in order to construct a network from data. However, the cost of this method increases quadratically with the size of the network and can be computation and memory heavy, with a time and space complexity of $O(N^{2})$. This restriction may be overcome using a sampling strategy, whereby only the connections between a portion of the nodes in each minibatch are learned.
- **Graph Convolution Module**: This module aims to describe spatial relationships in a network by incorporating knowledge from a node’s neighbors. It comprises two mix-hop propagation layers that handle the entrance and outflow of data independently through each node. The outputs of these two levels are combined to provide the net inflow information. The Graph Convolution Module aims to connect a node’s information with its neighbors. In our case, since the nodes represent the POIs visited at that specific moment in time, and the neighbors of each node describe the next location visited starting from the previous node, this fuse operation should improve the understanding of the model on how the locations are distributed and the general trajectory of the users.
- **Temporal Convolution Module**: To extract high-level temporal information, this module uses a collection of common dilated 1D convolution filters. There are two dilated inception layers in this module. A filtering tangent hyperbolic activation function follows the first dilated inception layer. The second dilated inception layer is followed by a sigmoid activation function, which serves as a gate to regulate how much data the filter may propagate to the subsequent module.
- **Output Module**: Composed by two convolution layers, transforming the channel dimension of the inputs to the desired output dimension, which for next location prediction, corresponds to setting the desired output dimension to one.

We used the same training procedure that was used for training the Flashback-LSTM models, i.e., the modes were trained for 100 epochs using the Cross-Entropy Loss Function. The Adam optimizer was used with a dynamic learning rate (modified every 20 epochs).

### 4.3. Temporal Fusion Transformer (TFT)

Our GPT model was based on the Temporal Fusion Transformer (TFT) architecture proposed by Lim et al. [40]—initially developed for multihorizon time series forecasting. The architecture includes a sequence-to-sequence layer to process known and observed inputs locally, static covariate encoders to encode context vectors for use elsewhere in the network, sample-dependent variable selection to reduce the impact of irrelevant inputs, and a temporal self-attention decoder to learn any long-term dependencies present in the dataset.

**Figure 4:**
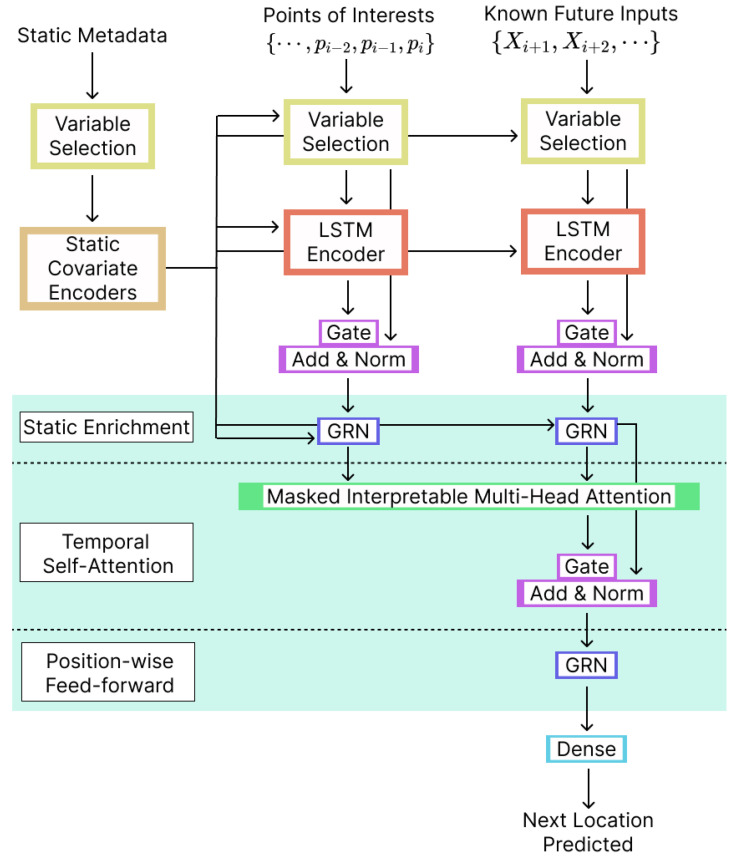
Architecture overview. Temporal Fusion Transformer for next-POI prediction.

A high-level overview of the TFT architecture for next-POI prediction is presented in Figure 4. There are three types of inputs for the model, Static Metadata, Past Inputs (which correspond to previous POIs), and Known Future Inputs (that can be extracted from the dataset in order to help with the predictions). The Known Future Inputs can be the day of the week and the time of the day.

Other components included in the TFT architecture are the following:

- **Gating Mechanism**: Used to skip over any parts of the architecture that are not being used by the model, offering adjustable depth and network complexity to support a variety of datasets and scenarios. The gating mechanism is implemented using a Gated Residual Network.
- **Variable Selection Networks**: TFT is a model that can manage several variables, but it is frequently uncertain what each variable specifically contributes to the result and how relevant it is. TFT uses Variable Selection Networks to select pertinent variables for static and time-dependent covariates individually for each occurrence. In addition to assisting with the identification of factors that are most important for the prediction task, this enables TFT to exclude irrelevant inputs that could have a negative effect on performance. This component is based on the Gated Residual Network and an external context vector obtained from a Static Covariate Encoder.
- **Static Covariate Encoder**: The TFT uses context vectors to encode static metadata. The vectors are produced by encoders based on Graph Recurrent Networks. These context vectors are a crucial component of the temporal fusion decoder’s processing of static variables.
- **Multihead Attention**: The TFT uses a self-attention mechanism, which is adapted from multihead attention in transformer-based architectures to learn long-term associations across various time steps. This component learns attention weights to estimate the significance of the different inputs.
- **Temporal Fusion Decoder**: This component learns the temporal connections contained in the dataset through a succession of layers.
  – *Locality Enhancement with the Sequence-to-Sequence Layer*: This layer aims to estimate informative segments (e.g., check-ins) in the time series data. Depending on the data, informative data points can be abnormalities, change points, or repetitive patterns.
  – *Static Enrichment Layer*: Because static covariates often substantially impact temporal dynamics, this layer incorporates static metadata and enhances temporal features.
  – *Temporal Self-Attention Layer*: After static enrichment, self-attention is applied, followed by a multihead attention mechanism at each time step. Decoder masking is then added to the multi-head attention layer to guarantee that each temporal dimension focuses on features that are in the past (and not in the future). The self-attention layer enables TFT to recognize long-range relationships that would be difficult for RNN-based systems to learn while maintaining causal information flow via masking.
  – *Position-Wise Feed-Forward Layer*: Applies a nonlinearity to the temporal self-attention layer’s outputs. Additionally, it contains a gated residual connection that provides a direct connection to the sequence-to-sequence layer by skipping over the full transformer block.
- **Output layer**: In order to generate the next-location predictions, we used a modified version of quantile forecasts. The outputs were generated using linear transformation of the output from the temporal fusion decoder.

We used the same training procedure that was used for training the other model types, i.e., the modes were trained for 100 epochs using the Cross-Entropy Loss Function. The Adam optimizer was used with a dynamic learning rate (modified every 20 epochs).

## 5. Experiments

### 5.1. Experimental Setup

#### 5.1.1. Hardware

All experiments were performed on the Ubuntu 22.04.1 server equipped with two GPU units, the NVIDIA GeForce RTX 3080 with 10 GB of memory, and the NVIDIA RTX A5000 with 24 GB of memory.

#### 5.1.2. Dataset Splits

It is important to note that since we were working with spatiotemporal data, we could not randomly shuffle the datasets. Otherwise, the models would be trained with events that are happening in the future. Consequently, we first sorted each dataset based on the timestamps, and then we created the following three splits:

- *Training set*: Used to train the predictive models and corresponds to the first 70% of the sorted datasets.
- *Test set*: Used to perform final model evaluation. This subset corresponds to the final 10% of the sorted datasets.
- *Validation set*: Used to validate the predictive models during training. This subset corresponds to 20% of the overall data. In temporal terms, 20% of the data were selected from the period between the training set and the testing set, i.e., from 70% to 90%.

In addition to the typical training/testing/validation splits, we also performed experiments with several subsets extracted from the original datasets. Both datasets (especially the Foursquare dataset) cover diverse populations (e.g., from Figure 2 it can be seen that Foursquare users come from all around the world). To investigate the behavior of the models for the different population-based subgroups, we first split the datasets into population-based datasets (e.g., based on users’ country, city, gender, and number of Twitter followers) and then split the resulting subdatasets into training/testing/validation datasets using the initial procedure (70%–10%–20% splits).

Section 5.2 presents the results for the overall datasets. Section 5.3 presents the results for the Foursquare subdataset, and Section 5.4 presents the results for the Gowalla subdatasets.

#### 5.1.3. Model Evaluation

For all models, the number of training epochs was set to 100 epochs. The final models were evaluated using the top-k Accuracy score (ACC@k). The ACC@k represents the ratio of the number of times the correct location was among the top-k output locations (ordered from most likely to least likely) divided by the total number of predictions. For example, for the ACC@3 metric, the target (correct location) is checked against a vector of the top 3 most likely output locations. The prediction is accurate (or a true positive) if the target is one of the top 3 vector elements. In the end, the total number of predictions is divided by the number of true positives.

### 5.2. Results—Full Datasets

Table 1 presents the results for next-place prediction for the three models and for each of the three datasets. The table shows that regardless of the type of model, the accuracy scores highly depend on the dataset. On the Breadcrumbs dataset, all models achieved an accuracy of higher than 0.94 (e.g., ACC@1). However, for the Foursquare and Gowalla datasets, the ACC@1 was lower than 0.27. These results are in line with related work [18,41]. Data sparsity is the main reason for the difference in accuracy scores between the sparse datasets (Foursquare and Gowalla) and the dense dataset (Breadcrumbs). This is because the Breadcrumbs dataset has a 15-min sampling frequency, i.e., the models provide a prediction for every 15-min slot, and there is a high probability that the users are at the same place. On the other hand, Foursquare and Gowalla are check-in-based datasets, and the users are rarely at the sample place twice in a row.

Regarding the accuracy score for each model, the Flashback-LSTM model—a state-of-the-art model from the related work specifically designed for next-place prediction on sparse datasets [18]—slightly outperformed the MTGCN and the TFT models. More specifically, on the Foursquare dataset, Flashback-LSTM achieved 0.2678 ACC@1, MTGCN achieved 0.2356 ACC@1, and TFT achieved 0.2577 ACC@1. Thus, the difference was less than 0.0322 (or 3.22 percentage points—p.p.). On the Gowalla dataset, the difference was less than 3.49 p.p. On the Breadcrumbs dataset, all models performed similarly, achieving an ACC@1 score of 0.94.

**Figure 5:**
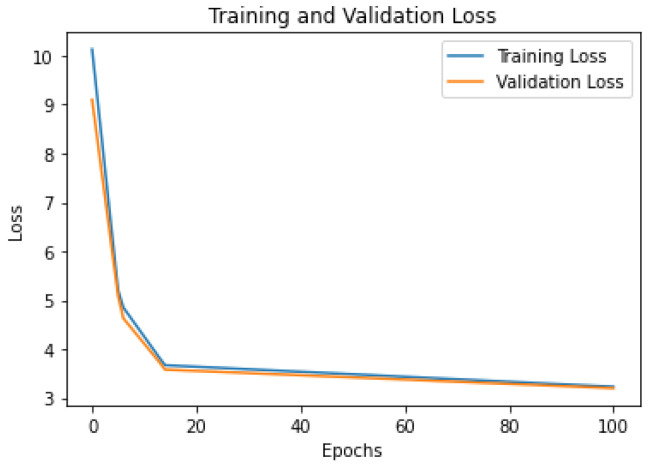
Flashback-LSTM model loss curves. Foursquare dataset.

**Figure 6:**
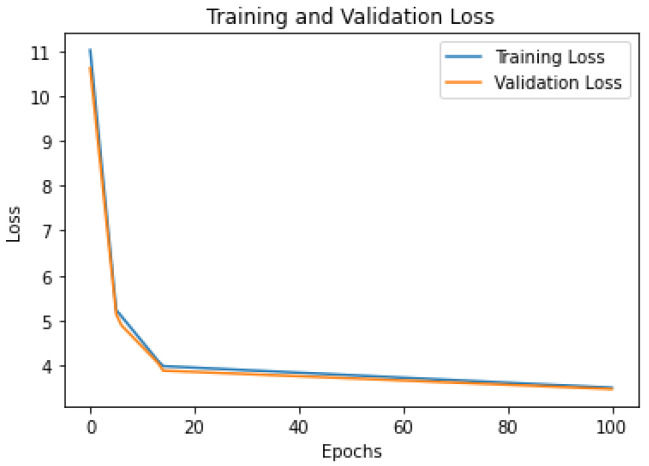
MTGCN model loss curves. Foursquare dataset.

**Figure 7:**
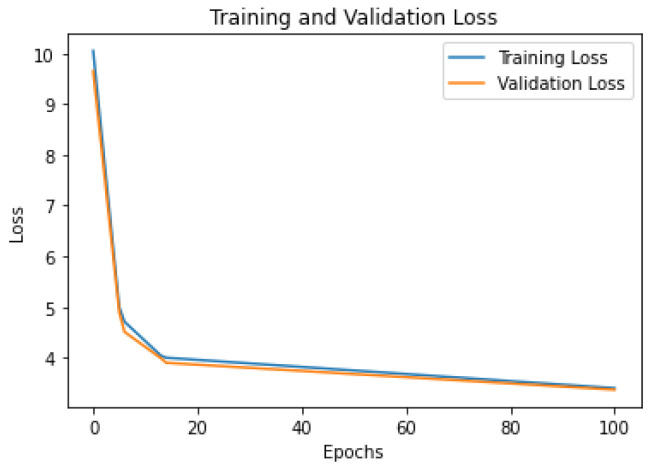
TFT model loss curves. Foursquare dataset.

To provide more insight into the training process of the models, we present the learning curves for the three models on the Foursquare dataset: Figure 5 shows the Flashback-LSTM model; Figure 6 shows the MTGCN model; and Figure 7 shows the TFT model. From the figures, it can be seen that (i) all models have similar learning patterns, i.e., the loss values (categorical cross-entropy) decreased significantly in the first twenty learning epochs. After that, the loss values decreased steadily at a lower rate; (ii) there were no abrupt variations in the learning curves, which signifies a stable learning process and consequently stable models; and (iii) the training loss values were close to the validation loss values throughout the overall training process, which signifies that all models avoided overtraining.

### 5.3. Results—Foursquare Subdatasets

We performed additional experiments on the Foursquare dataset to evaluate the models more thoroughly. The Foursquare dataset contains user profile data in addition to check-in data. Using the user profile information, we created seven subdatasets based on the users’ country, city, gender, and number of Twitter followers. Information about the size of the subdatasets is presented in Table 2:

1. *_US*: Data from US-based users.
2. *_NYC*: Data from New York City users.
3. *_TKY*: Data from Tokyo users.
4. *_NYC_gender*: Data from male users from New York City. We chose males because the number of male users was higher than the number of female users.
5. *_TKY_gender*: Data from male users from Tokyo. We chose males because the number of male users was higher than the number of female users.
6. *_NYC_gender*: Data from the top 1000 New York City users ranked by the number of Twitter followers.
7. *_TKY_gender*: Data from the top 1000 Tokyo users ranked by the number of Twitter followers.

These subdatasets enabled us to evaluate the behavior of the models with a varying number of users (from 480 up to 65,340), a varying number of POIs (from 392 up to 61,794), and a varying number of training instances/check-ins (from 45,510 up to 8,180,051).

The results of the experiments are presented in Table 3. From the table, it can be seen that the results follow a similar trend as the results from the previous subsections, i.e., the full dataset experiments (Table 1): (i) Regardless of the type of model, the accuracy scores depend strongly on the dataset; (ii) the Flashback-LSTM model slightly outperformed the MTGCN and the TFT models. More specifically, if we look at ACC@1 scores, the difference between Flashback-LSTM and TFT was 0 to 2.5 p.p, and the difference between Flashback-LSTM and MTGCN was between 1 and 3.5 p.p.; and (iii) the TFT model slightly outperformed the MTGCN in all experiments.

### 5.4. Results—Gowalla Subdatasets

With the Gowalla dataset, we were not able to create diverse subdatasets (as we did with the Foursquare subdatasets), because Gowalla does not contain user profile data, except for the number of friends for each user. Thus, we created a Gowalla subdataset by taking the top 1000 users ranked by their number of friends. The resulting subdataset (*Gowalla_firends*) is presented in Table 4.

The results of the experiments are presented in Table 5. On the *Gowalla_firends* subdataset, Flashback-LSTM achieved the highest ACC@1 score of 0.4, whereas both the MTGCN and the TFT model achieved an ACC@1 score of 0.36.

## 6. Discussion and Conclusions

### 6.1. Discussion of the Results

We presented a study that explored deep learning models based on GPT and GCNs for mobility modeling. More specifically, we developed models for next-place prediction, and we evaluated them using two sparse datasets (Foursquare and Gowalla) and one dense dataset (Breadcrumbs). Including the eight subdatasets we created from the Foursquare and the Gowalla datasets, we performed experiments on eleven different datasets. Some general findings that were confirmed in all eleven experiments are the following:

- Regardless of the type of model, the accuracy scores were highly dependent on the dataset. Data sparsity was the main reason for the difference in accuracy scores between the sparse datasets (Foursquare and Gowalla) and the dense dataset (Breadcrumbs).
- For the sparse datasets, Flashback-LSTM—specifically designed for next-place prediction on sparse datasets—slightly outperformed the GPT-based and GCN-based models. This indicates that some of the mechanisms that are specific to the Flashback-LSTM could be useful to improve the quality of the other models. For instance, Flashback-LSTM explicitly takes advantage of spatiotemporal context in the RNN layers to seek past hidden states with good predictive potential. By performing “flashbacks” on the RNN’s hidden states, the model may assess how relevant the current input is in light of analogous prior scenarios.
- For the dense dataset, all the models achieved equal evaluation scores (see Table 1).
- The TFT model slightly outperformed the MTGCN in all experiments.

The learning curves of the three models revealed that all the models have similar, stable learning processes, and all models avoided overtraining, i.e., there were no substantial differences between training and the validation learning curves of the models.

The experimental results showed that GPT- and GCN-based architectures are a promising direction for future mobility modeling, especially given that future use cases will likely involve dense datasets provided by ubiquitous sensing devices.

### 6.2. Data Privacy

The methods presented in this study are fully centralized, i.e., we assume that the data from all users are available at one data center. However, such a centralized approach raises serious privacy concerns, given the sensitivity of mobility data. Since location and movement data are included in the definition of personal data, the EU General Data Protection Regulation (GDPR) has highlighted the significance of this information [42]. In the real world, there are currently no generally applicable methods for service providers to track users without seriously jeopardizing their right to privacy (see, for example, [1,43,44]). Promising privacy-aware approaches that can be explored in combination with GPT and GCN models include

- The use of federated learning (FL) instead of centralized learning for training the predictive models. FL allows devices to learn a shared model collaboratively while keeping all the training data on-device [45]. While offering promising privacy-aware solutions, FL is a relatively new approach with many open challenges, including scalability and federated model training and evaluation [41].
- The use of cohort-based modeling [38], whereby, instead of estimating the behavior of each user separately, cohort-based models aim to estimate the behavior of a group of similar users (cohorts). Thus, the specific users are removed from the focus of the processing pipelines. Cohort-based models treat each user’s data as just one data point that is anonymously aggregated within the pool of cohort data. Such an approach provides k-anonymity by default (k is the size of the cohort) [46], enabling privacy-aware analytics.

### 6.3. Limitations and Future Work

State-of-the-art models, including GPT and GCN models, rely on large datasets to learn patterns and make predictions. These datasets can be biased or incomplete. For instance, the model may be less accurate in predicting outcomes for some groups if the dataset used to train the model does not include data for some specific regions or demographic categories. Additionally, factors, such as one’s economic situation and educational background, may also need to be considered when estimating mobility patterns, and it is unclear how models such as GPT and GCN models would be able to include such information. Furthermore, GPT and GCN models are complex and challenging to interpret, which makes it difficult for policymakers and stakeholders to evaluate and validate the predictions made by the model. Thus, Explainable AI techniques may be required to make these approaches more applicable (e.g., [47]). In addition to transparency and privacy, fairness is a third dimension that needs to be considered to avoid discriminatory outcomes. Finally, to capture the dynamic nature of human mobility, such models will need to be updated frequently to maintain their predictive power.

In this study, we did not perform a thorough hyperparameter optimization, which could be a promising direction for improving the evaluation scores of the GPT- and GCN-based models. Note that the Flashback architecture was optimized to work with sparse datasets in the original study. Given the large number of inter-related hyperparameters and the overall complexity of the DL architectures, a hyperparameter search based on Bayesian optimization may be an appropriate approach to solve this problem [48].

## Figures and Tables

**Table 1:** Top-k Accuracy scores (k = 1, 5, and 10) for the three models for each of the three datasets (Foursquare, Gowalla, and Breadcrumbs).

	Flashback-LSTM				MTGCN			TFT	
	**ACC@1**	**ACC@5**	**ACC@10**	**ACC@1**	**ACC@5**	**ACC@10**	**ACC@1**	**ACC@5**	**ACC@10**
Foursquare	0.2678	0.5536	0.6326	0.2356	0.4928	0.6251	0.2577	0.5389	0.6212
Gowalla	0.1281	0.2935	0.3637	0.0932	0.2628	0.3421	0.1076	0.271	0.3572
Breadcrumbs	0.9477	0.9839	0.9931	0.9442	0.9786	0.992	0.947	0.9821	0.9928

**Table 2:** Size of the Foursquare dataset and the corresponding subdatasets based on the city, gender, and number of Twitter followers.

Info	4sq	_US	_NYC	_TKY	_NYC_gender	_TKY_gender	_NYC_twitt	_TKY_twitt
Checkins	8,180,051	678,663	281,374	802,344	196,223	45,510	49,803	217,381
Users	65,340	11,116	4214	4059	2876	480	1000	1000
POIs	61,794	7183	2956	4180	2104	392	545	1362

**Table 3:** Top-k Accuracy scores (k = 1, 5, and 10) for the three models on seven Foursquare subdatasets based on the city, gender, and number of Twitter followers.

	Flashback LSTM				MTGCN			TFT	
	**ACC@1**	**ACC@5**	**ACC@10**	**ACC@1**	**ACC@5**	**ACC@10**	**ACC@1**	**ACC@5**	**ACC@10**
4sq	0.2678	0.5536	0.6326	0.2356	0.4928	0.6251	0.2577	0.5389	0.6212
4sq_US	0.3946	0.8016	0.8723	0.3531	0.7884	0.861	0.3756	0.7692	0.8711
4sq_NYC	0.4031	0.8112	0.8802	0.3588	0.7979	0.8752	0.3841	0.8013	0.8749
4sq_NYC_gender	0.4065	0.8304	0.9062	0.361	0.8072	0.8824	0.3858	0.8136	0.8909
4sq_NYC_tweet	0.4495	0.855	0.9385	0.4139	0.8221	0.9264	0.4243	0.8348	0.9215
4sq_TKY	0.2245	0.5301	0.6284	0.2062	0.5059	0.5928	0.2228	0.5284	0.6193
4sq_TKY_gender	0.3483	0.7378	0.8521	0.3229	0.7091	0.8133	0.3401	0.7226	0.8432
4sq_TKY_tweet	0.2589	0.6032	0.7089	0.2483	0.5812	0.6817	0.2498	0.5941	0.6916

**Table 4:** Size of the Gowalla dataset and the corresponding subdataset based on the number of friends each user has.

Info	Gowalla	Gowalla_friends
Checkins	6,442,890	196,591
Users	196,591	1000
POIs	35,639	504

**Table 5:** Top-k Accuracy scores (k = 1, 5, and 10) for the three models on the Gowalla subdataset.

	Flashback LSTM				MTGCN			TFT	
	**ACC@1**	**ACC@5**	**ACC@10**	**ACC@1**	**ACC@5**	**ACC@10**	**ACC@1**	**ACC@5**	**ACC@10**
Gowalla	0.1281	0.2935	0.3637	0.0932	0.2628	0.3421	0.1076	0.271	0.3572
Gowalla_friends	0.4022	0.7966	0.871	0.3597	0.6923	0.8015	0.3616	0.7052	0.8124

## Data Availability

The study used publicly available datasets. The details are presented in the section Datasets.
